# Multiple splice variants within the bovine silver homologue (*SILV*) gene affecting coat color in cattle indicate a function additional to fibril formation in melanophores

**DOI:** 10.1186/1471-2164-8-335

**Published:** 2007-09-24

**Authors:** Christa Kuehn, Rosemarie Weikard

**Affiliations:** 1 Research Institute for the Biology of Farm Animals (FBN), Res. Unit Molecular Biology, Wilhelm-Stahl-Allee 2, D-18196 Dummerstorf, Germany

## Abstract

**Background:**

The silver homologue(*SILV*) gene plays a major role in melanosome development. *SILV *is a target for studies concerning melanoma diagnostics and therapy in humans as well as on skin and coat color pigmentation in many species ranging from zebra fish to mammals. However, the precise functional cellular mechanisms, in which *SILV *is involved, are still not completely understood. While there are many studies addressing *SILV *function upon a eumelaneic pigment background, there is a substantial lack of information regarding the further relevance of *SILV*, e.g. for phaeomelanosome development.

**Results:**

In contrast to previous results in other species reporting *SILV *expression exclusively in pigmented tissues, our experiments provide evidence that the bovine *SILV *gene is expressed in a variety of tissues independent of pigmentation. Our data show that the bovine *SILV *gene generates an unexpectedly large number of different transcripts occurring in skin as well as in non-pigmented tissues, e.g. liver or mammary gland. The alternative splice sites are generated by internal splicing and primarily remove complete exons. Alternative splicing predominantly affects the repeat domain of the protein, which has a functional key role in fibril formation during eumelanosome development.

**Conclusion:**

The expression of the bovine *SILV *gene independent of pigmentation suggests *SILV *functions exceeding melanosome development in cattle. This hypothesis is further supported by transcript variants lacking functional key elements of the *SILV *protein relevant for eumelanosome development. Thus, the bovine *SILV *gene can serve as a model for the investigation of the putative additional functions of *SILV*. Furthermore, the splice variants of the bovine *SILV *gene represent a comprehensive natural model to refine the knowledge about functional domains in the SILV protein. Our study exemplifies that the extent of alternative splicing is presumably much higher than previously estimated and that alternatively spliced transcripts presumably can generate molecules of deviating function compared to their constitutive counterpart.

## Background

The silver homologue(*SILV*) gene has been a target for many investigations concerning development of melanosomes, which are the specific pigment carrying compartments within melanophores, e.g. in melanocytes. In humans, *SILV *plays a major role in studies regarding melanoma diagnosis and therapy, because SILV is a sensitive melanoma marker on transcript and protein level [1,2] and represents a melanoma specific antigen recognized by tumor infiltrating cytotoxic T lymphocytes [3]. The SILV protein also known as PMEL17, GP100 or ME20 [4] is crucial for proper formation and maturation of melanosomes. In stage II melanosomes, processed SILV protein aggregates to form fibrils, to which presumably the eumelanin pigment is attached [5]. Due to its role in melanosome development, SILV has also been subject to several studies investigating the genetic background of coat color.

Coat color phenotype in mammals is dependent on a series of genes determining distribution of melanocytes (e.g. *KIT*, [6]), synthesis of the two essential pigments, eumelanin (black) and phaeomelanin (red) (e.g. *MC1R*, [7]), and intra- and intercellular transport mechanisms of proteins relevant for coat color expression (e.g. *MATP*, [8]). In cattle, variants in the *MC1R *gene result in exclusively eumelaneic (black) or phaeomelaneic (red) skin [9]. Spotted individuals exhibit delimited, white skin areas lacking melanocytes similar to piebald mice [10]. Similar to other species, dilution loci resulting in a diluted type of the original coat color are known in cattle, e.g. the *Dilution *locus in the Charolais breed (Dc). The putative role of *SILV *for various coat color dilution loci was described in a number of species, including mouse, dog, chicken, horse, and cattle [11-16]. There is concurring indication that concerning coat color the effect of a mutation in the *SILV *gene in mouse, horses and dogs seems to be restricted to the dilution of eumelaneic pigment. This is underlined by experiments in mice indicating that *SILV *expression seems to be restricted to melanocytes expressing eumelanin [17]. In cattle, however, there is still some controversy regarding the potential role of SILV in phaeomelanosome development, because up to now there is no unequivocal experimental evidence rejecting or propagating SILV mutations as causal background for phaeomelanin dilution [14,18]. First reports on splice variants for the human *SILV *gene [19] and a retroposon insertion in intron 10 of the canine *SILV *gene that seems to affect the correct splicing of the gene in *merle *dogs [12] raise the question whether putative splice variants of the *SILV *gene may be specifically involved in melanosome development. Comprehensive studies in humans revealed that alternative splicing is a frequent mechanism altering spatial expression pattern and function of proteins [20,21].

In the present study, we present a comprehensive description of the complex expression pattern of the bovine *SILV *gene in pigmented and non-pigmented tissues. Multiple transcript variants affecting functional key domains of the SILV protein indicate that the bovine *SILV *gene may serve as a model for investigations about alternative splicing to generate molecules of obviously deviating function compared to constitutive transcripts.

## Results

### Identification of the *SILV *transcription start site

**Figure 1:**
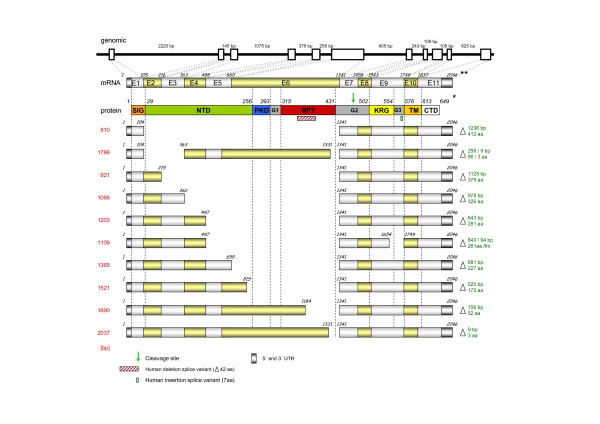
**Structure of the bovine *SILV *mRNA including alternative transcripts**. Position of exons (open boxes) and introns (solid line) in the *SILV *genomic sequence are indicated. Protein domains according to [4,31] are shown: SIG: signal peptide, NTD: N-terminal domain, PKD: polycystic kidney domain, RPT: repeat domain, KRG: Kringle-like domain, TM: transmembrane domain, CTD: C-terminal domain, G1, G2, G3: undefined domains. E: *SILV *exon.* Numbers in the SILV protein diagram represent the first amino acid of the respective domain. ** Numbers for the *SILV *transcripts represent the first nucleotide for the respective exon or the last constitutive nucleotide of an alternative transcript. fm: frame shift mutation. Length [in bp] of the alternative c *SILV *transcripts is indicated in red (left), the missing part relative to the constitutive *SILV *transcript (nucleotides and amino acids) is indicated in green (right).

Analysis of several 5'RACE clones from total RNA of eumelaneic, non-dilute (black) skin indicated a sharp peak of transcription start sites (TSS) at position -28 bp to the A of the translation start ATG of the *SILV *gene (GenBank: EF065525). No further promoters were detected. The *SILV *transcription start obtained in this study adds an additional 7 bp to the previously deposited bovine *SILV *cDNA sequence [14]. The cDNA sequence generated by this experiment based on CAP carrying full length mRNA confirmed previous findings about the structure of the bovine *SILV *gene comprising 2046 bp organized in 11 exons (Figure 1) and encoding 649 amino acids. Aligning the obtained *SILV *cDNA sequence with the bovine genomic contig NCBI: NW_001495046 [22]) indicated that the bovine *SILV *gene spans a total of 8107 bp with introns sizes ranging from 108 to 2220 bp. A TTATA motif representing the putative TATA box of the *SILV *promoter is located 30 bp upstream of the transcription initiation site.

### Detection of splice variants

**Figure 2:**
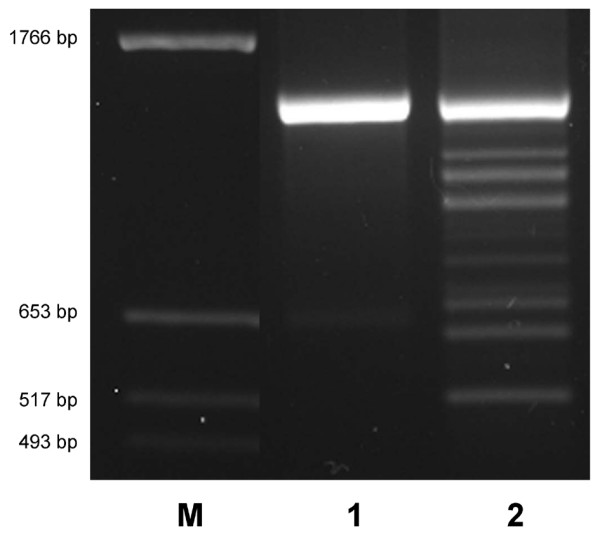
**RT-PCR products in the *SILV *gene in eumelaneic black bovine skin**. RT-PCR products were generated from pigmented, eumelaneic (black) bovine skin with primers spanning *SILV *exon 1 to exon 7 (E1_F3_/E7_R1_). M: DNA marker, 1: cloned constitutive *SILV *cDNA, 2: cDNA from eumelaneic (black), non-dilute (*Dc*^*d*^*/Dc*^*d*^) skin

Multiple RT-PCR fragments were detected when amplifying *SILV *cDNA from RNA of eumelaneic (black), non-diluted skin with primers in the 5' and 3' UTR of the *SILV *gene (5UTR_F1 _– E11_R2_; Table 1, 2). This diversity of transcripts was also obtained with a primer combination spanning exon 1 to exon 7 (E1_F3 _– E7_R1_) of the bovine *SILV *gene (Figure 2). While the constitutive transcripts (2039 bp or 1430 bp, respectively, confirmed by sequencing) were dominating, further fragments up to 900 bp smaller than the constitutive transcript were identified with both primer pairs. In contrast, cloned *SILV *constitutive cDNA yielded only the expected 2039 or 1430 bp PCR fragment, respectively.

**Table 1:** Primers for reverse transcription and amplification in the bovine *SILV *gene

Name	Position^a^	Exon	Sequence (5' – 3')
E1_F3_	69	1	TGATGGGTGTTCTTCTGGCTG
E3_F1_	280	3	CTCTATTGCCTTGCACTTTCC
E3_R1_	362	3	CATTGATGATGGTGTTGTTGG
E5_R1_	656	5	TAATGGTGAAGGCTGAACTGG
E5_F1_	575	5	AACATGGAAGTGACTGTCTACC
E7_R1_	1430	7	AGCCATAGCGATACAGAACAC
E7_F1_	1360	7	GGATGACACTGCCACCTTAG
E11_R2_	2046	11	AGGGAAGACCAGAGAAAAGAC
E6_F2_	952	6	CACTACAGATAGGCATGTGAC
E6_R2_	1030	6	GCCCATGACTTCTGTAGTAGG
E10_F1_	1773	10	CTCCTCTGTTCGTGGGCATC
E9_F1_	1643	9	GTTTTGCACCAGGTACTGAAG
RACE_E7N	1441	7	CAGGGTGAGGGAAAAGGAGCCATAG
5UTR_F1_	8	1	GTTGCTGGAAGGAAGAACAGG
SPex23_F1_	88	1/4	TGTAGGGACCACAGAAG**GGAG**
E5_R1_	656	5	TAATGGTGAAGGCTGAACTGG
SPex56_F1_	481	4/7	TGTCTGGAAGACCTGGG**GCT**
E9_R2_	1587	9	TGACACCCTGGCGATGAGATG
E5_F2_	565	5	GGGCACATATAACATGGAAGTG
SPex6_R1_	1358	7/5	GCAGGGGACTCAGGGAGC**CAG**
SPex6A_F1_	798	6/7	CCTACACCTGGGACTTTG**GCT**
SPex6B_F1_	1167	6/7	CAACTGCAAAAGCTACAG**GCT**
E7_F2_	1409	7	TGTGTTCTGTATCGCTATGGCTC
SPex9_R1_	1767	9/10	CTGAGGCCTGCTTCTTGCC**CTG**

^a ^Position within the bovine *SILV *mRNA (according to GenBank: EF065525). Underlined, bold sequence: nucleotides specific for alternative splice site.

**Table 2:** Regions of the bovine *SILV *mRNA tested for expression by RT-PCR in skin and non-pigmented body tissues

Primer combination	Region	Expected fragment [bp]	Observed fragment [bp]		
			Eumelaneic skin	Phaeomelaneic skin	Non-pigmented tissues
5UTR_F1 _– E11_R2_	exon 1 – exon 11	2039	2039, 1102 and multiple further fragments	n.a.	n.a.
E1_F3 _– E7_R1_	exon 1 – exon 7	1430	1430 and further multiple bands	n.a.	n.a.
E1_F3 _– E3_R1_	exon 1 – exon 3	294	294	294	n.a.
E3_F1 _– E5_R1_	exon 3 – exon 5	377	377	377	n.a.
E5_F1 _– E7_R1_	exon 5 – exon 7	856	856, 700, 331, 175	856, 700, 331, 175	856, 700, 331, 175 (pg, tg, ki, ag, li, lu, he, br, ru, if, sf, sf, mg, fu, co, je, mu)
E7_F1 _– E11_R2_	exon 7 – exon 11	687	687; 593	687; 593	n.a.
E6_F2 _– E7_R1_	exon 6 – exon 7	479	479, 324	479, 324	n.a.
E5_F1 _– E6_R2_	exon 5 – exon 6	456	456	456	n.a.
E9_F1 _– E11_R2_	exon 9 – exon 11	404	404	404	404 (pg, tg, ki, ag, li, lu, he, br, ru, if, sf, sf, mg, fu, co, je, mu)
E10_F1 _– E11_R2_	exon 10 – exon 11	274	274	274	n.a.
E1_F3 _– E5_R1_	exon 1 – exon 5	588	n.a.	n.a.	588 (pg, tg, ki, ag, li, lu, he, br, ru, if, sf, sf, mg, fu, co, je, mu)

pg: pituitary gland, tg: thyroid gland, ki: kidney, ag: adrenal gland, li: liver, lu: lung, he: heart, br: brain, ru: rumen, if: intestinal fat, sf: subcutaneous fat, pf: perirenal fat, mg: mammary gland, du: duodenum, co: colon, je: jejunum, mu: skeletal muscle. n.a.: not analyzed

**Figure 3:**
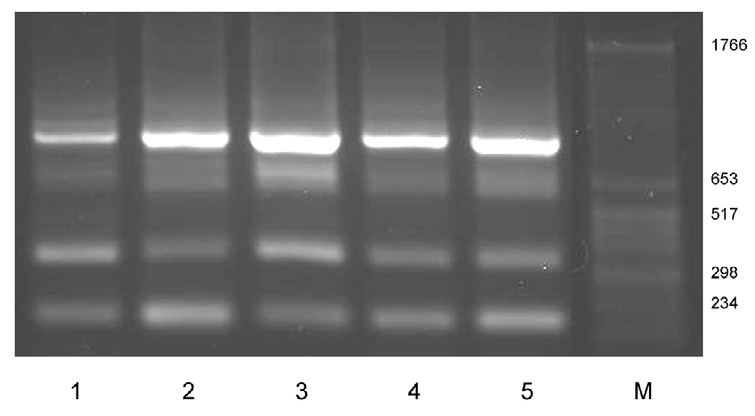
**Expression of *SILV *in eumelaneic and phaeomelaneic bovine skin**. RT-PCR products for the bovine *SILV *region spanning exon 5 to exon 7 were generated with primer E5_F1 _– E7_R1 _in differentially pigmented bovine skin. 1: non-pigmented skin from a heterozygous dilute (*Dc*^*D*^*/Dc*^*d*^), eumelaneic (*E*^*D*^*/E*^*D*^) spotted individual; 2: pigmented skin from a heterozygous dilute (*Dc*^*D*^*/Dc*^*d*^), eumelaneic (*E*^*D*^*/E*^*D*^) spotted individual; 3: crème white skin from a homozygous dilute (*Dc*^*D*^*/Dc*^*D*^), eumelaneic (*E*^*D*^*/E*^*D*^) non-spotted individual; 4: non-pigmented skin from a heterozygous dilute (*Dc*^*D*^*/Dc*^*d*^), phaeomelaneic (*E*^*e*^*/E*^*e*^) spotted individual; 5: pigmented skin from a homozygous non-dilute (*Dc*^*d*^*/Dc*^*d*^), eumelaneic (*E*^*D*^*/E*^*e*^) spotted individual; M: marker

A series of PCR amplifications in *SILV *cDNA samples with primers dissecting the transcribed *SILV *sequence into smaller segments enabled a better discrimination of the generated fragments. Exons 6, 8 and 9 were identified as putative regions for alternative splicing events, because at least two RT-PCR fragments could be unambiguously discriminated with primer combinations spanning exon 5 – 7 (E5_F1 _– E7_R1_) and exon 7 – 11 (E7_F1 _– E11_R2_). In contrast, only single RT-PCR fragments were observed for the primer combinations covering exon 1 – 3, exon 3 – 5, exon 5 – 6, exon 9 – 11 and exon 10 – 11. Notably, all identified fragments were present in all differentially pigmented skin samples investigated (Table 2, Figure 3). Our data show that the expression of the bovine *SILV *gene is not restricted to eumelaneic (black), non-dilute (*Dc*^*d*^*/Dc*^*d*^) skin, but also occurs in skin expressing exclusively phaeomelanin. Furthermore, also white skin sections of spotted individuals with eumelaneic or phaeomelaneic background showed *SILV *gene expression as well as the crème white skin of a eumelaneic homozygous dilute *Dc*^*D*^*/Dc*^*D *^individual.

The *SILV *transcripts additional to the constitutive fragment that had been identified by RT-PCR were isolated from the agarose gel and sequenced to reveal the specific DNA sequence of the different fragments. Alignment of the obtained sequences to the reference constitutive cDNA (GenBank: EF065525) showed that the additional *SILV *transcripts lacked different parts of the *SILV *mRNA (Figure 1). With primers spanning exon 5 to exon 7 (E5_F1 _– E7_R1_), three alternative transcripts (according to GenBank: EF065525) were characterized: Δ660–1340 lacking 681 bp corresponding to the entire exon 6, Δ816–1340 lacking 525 bp from the 3' end of exon 6 and Δ1185–1340 lacking 156 bp from the 3' end of exon 6. With primers spanning exon 7 to exon 11 (E7_R1 _– E11_R2_), we obtained an alternative transcript Δ1655–1748 lacking 94 bp from the 3' end of exon 9. Furthermore, sequencing of an additional RT-PCR transcript amplified with primers from exon 1 and exon 11 (5UTR_F1 _– E11_R2_) detected a *SILV *cDNA Δ498–1340/Δ1655–1748 lacking exon 5, exon 6 and 94 bp from the 3' end of exon 9 (Figure 1).

Diversity in *SILV *transcripts was also confirmed in the course of our 5'RACE experiments. Several clones containing inserts smaller than the expected size (1441 bp) were detected. Sequencing of the respective clones revealed *SILV *cDNAs starting from the identified transcription start, but devoid of distinct other regions in the remaining *SILV *mRNA. On the one hand, we received transcripts confirming already detected alternative splice variants e.g., Δ498–1340, Δ660–1340, Δ816–1340, and Δ1185–1340. Additionally, we identified two further internal splice sites generating a transcript Δ105–362 (skipping exons 2 and 3)/Δ1332–1340 (lacking 9 bp at the 3' end of exon 6 (Figure 1).

In order to obtain indication whether combinations of alternative splicing events occurred, we sequenced a collection of clones from a plasmid library containing *SILV *RT-PCR products of variable size generated by cDNA amplification of eumelaneic (black), non-dilute (*Dc*^*d*^*/Dc*^*d*^) skin with primers spanning exon 1 to exon 7 (E1_F3 _– E7_R1_). Alignment of the generated sequences to the *SILV *constitutive transcript confirmed previously detected transcripts and yielded an additional new series of transcripts (Figure 1) with deleted exons: Δ363 – 1340 (lacking exon 4 – 6), Δ216 – 1340 (lacking exon 3 – 6) and Δ105 – 1340 (lacking exon 2 – 6).

Only some alternative internal splice sites completely conformed to the GT-AG rule for splice donor and splice acceptor sites. Notably, the alternative splice sites did not affect the reading frame of the transcript except for Δ498–1340/Δ1655–1748. In the transcript Δ498–1340/Δ1655–1748, a premature stop codon is generated at position c.1805.

### Confirmation of *SILV *splice variants in differentially pigmented skin

**Figure 4:**
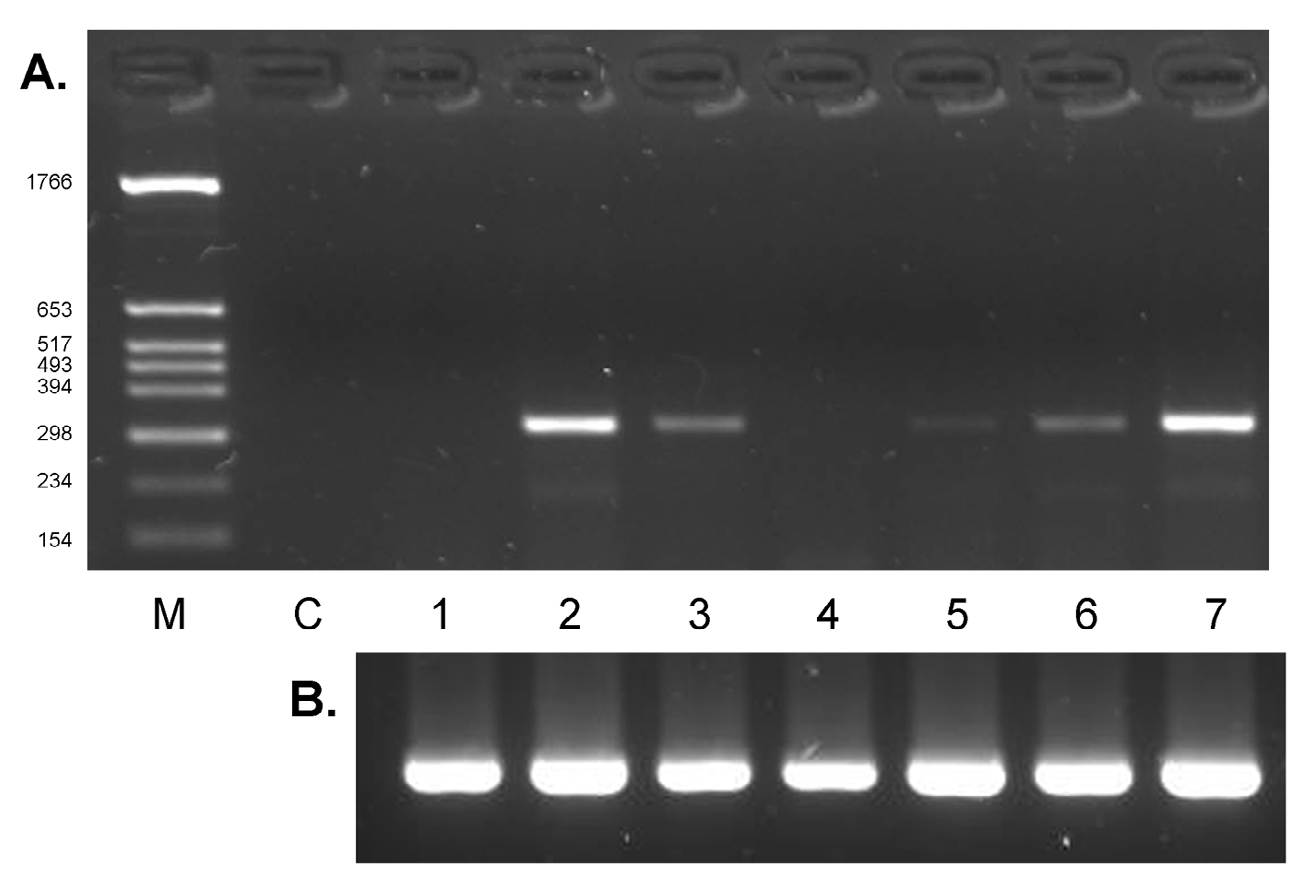
**Splice site specific *SILV *RT-PCR in differentially pigmented bovine skin**. A. Detection of bovine *SILV *splice variant Δ105–362 in differentially pigmented skin. RT-PCR products generated by splice site specific RT-PCR with primers SPex23_F1 _– E5_R1_. M: marker; C: negative control, 1: non-pigmented skin from a heterozygous dilute (*Dc*^*D*^*/Dc*^*d*^), eumelaneic (*E*^*D*^*/E*^*D*^) spotted individual; 2: pigmented skin from a heterozygous dilute (*Dc*^*D*^*/Dc*^*d*^), eumelaneic (*E*^*D*^*/E*^*D*^) spotted individual; 3: crème white skin from a homozygous dilute (*Dc*^*D*^*/Dc*^*D*^), eumelaneic (*E*^*D*^*/E*^*D*^) non-spotted individual ; 4: non-pigmented skin from a heterozygous dilute (*Dc*^*D*^*/Dc*^*d*^), phaeomelaneic (*E*^*e*^*/E*^*e*^) spotted individual; 5: pigmented skin from a heterozygous dilute (*Dc*^*D*^*/Dc*^*d*^), phaeomelaneic (*E*^*e*^*/E*^*e*^) spotted individual; 6: non-pigmented skin from a homozygous non-dilute (*Dc*^*d*^*/Dc*^*d*^), eumelaneic (*E*^*D*^*/E*^*e*^) spotted individual; 7: pigmented skin from a homozygous non-dilute (*Dc*^*d*^*/Dc*^*d*^), eumelaneic (*E*^*D*^*/E*^*e*^) spotted individual. B. RT-PCR products generated with GAPDH primers.

In order to confirm alternative splice sites in the bovine *SILV *gene, we developed splice variant specific RT-PCR tests for an exemplary subset of the detected splice variants (Table 3, Figure 4). Splice variant profiling revealed the alternative splice variants Δ498–1340, Δ816–1340 and Δ1185–1340 in all differentially pigmented skins (Table 4). In contrast, we obtained a specific pattern for Δ105–362, which was not detected in non-pigmented, white skin of spotted individuals heterozygous for the dilute locus (*Dc*^*D*^*/Dc*^*d*^) regardless whether on a eumelaneic or a phaeomelaneic background. Notably, non-pigmented white skin of a spotted, homozygous non-dilute (*Dc*^*d*^*/Dc*^*d*^) individual exhibited this splice site albeit at a lower level than the pigmented counterpart. Skin with a crème white coat color characteristic for individuals homozygous at the dilution locus *Dc*^*D*^*/Dc*^*D *^also displayed the alternative splicing Δ105-362. It has to be noted that crème white skin has an essentially pigmented background with melanocytes [23], however, pigmentation is diluted to almost invisibility. In contrast, non-pigmented skin of spotted or piebald individuals is devoid of melanocytes resulting in a white coat color [10].

**Table 3:** Splice site specific RT-PCR primer combinations applied for the detection of alternative bovine *SILV *transcripts

Alternative splice site	Upstream primer	Downstream primer	Expected alternative transcript [bp]
Δ105–362	SPex23_F1 _TGTAGGGACCACAGAAG**GGAG**	E5_R1 _TAATGGTGAAGGCTGAACTGG	311
Δ498–1340	SPex56_F1 _TGTCTGGAAGACCTGGG**GCT**	E9_R2 _TGACACCCTGGCGATGAGATG	264
Δ660–1340	E5_F2 _GGGCACATATAACATGGAAGTG	SPex6_R1 _GCAGGGGACTCAGGGAGC**CAG**	113
Δ816–1340	SPex6A_F1 _CCTACACCTGGGACTTTG**GCT**	E9_R2 _TGACACCCTGGCGATGAGATG	265
Δ1185–1340	SPex6B_F1 _CAACTGCAAAAGCTACAG**GCT**	E9_R2 _TGACACCCTGGCGATGAGATG	265
Δ1655–1748	E7_F2 _TGTGTTCTGTATCGCTATGGCTC	SPex9_R1 _CTGAGGCCTGCTTCTTGCC**CTG**	265

Underlined, bold sequence: nucleotides specific for alternative splice site.

**Table 4:** Expression of bovine *SILV *splice variants by splice site specific RT-PCR in differentially pigmented skin and non-pigmented tissues

Tissues	Δ105–362	Δ498–1340	Δ660–1340	Δ816–1340	Δ1185–1340	Δ1655–1748
Non-pigmented, heterozygous dilute, eumelaneic	-	+	+	+	++	+
pigmented, heterozygous dilute, eumelaneic	++	++	+	++	++	++
pigmented, homozygous dilute, eumelaneic	+	++	+	++	++	++
Non-pigmented, heterozygous dilute, phaeomelaneic	-	+	+	+	++	+
Pigmented, heterozygous dilute, phaeomelaneic	+	++	+	++	++	++
Non-pigmented, homozygous non-dilute, eumelaneic	+	++	+	++	++	++
Pigmented, homozygous non-dilute, eumelaneic	++	++	+	++	++	++
						
liver	-	-	-	+	-	-
duodenum	-	-	-	+	-	-
pituitary gland	-	-	-	+	-	-
lung	-	+	+	+	-	-
heart	-	-	-	+	-	-
kidney	-	+	-	+	-	+
mammary gland	-	+	-	+	+	+

-: no expression detected, + expression detected, ++ enhanced expression detected

### Analysis of tissue specific *SILV *gene expression

**Figure 5:**
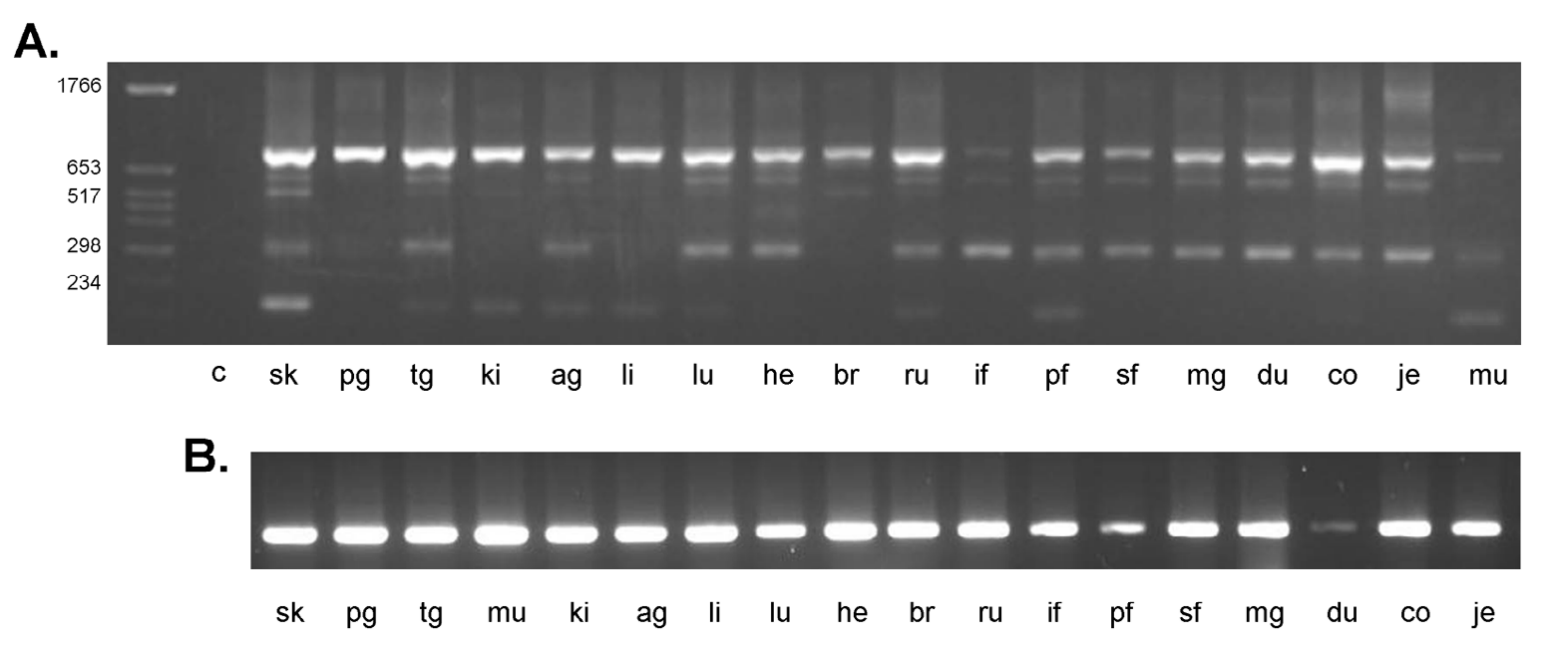
**Expression of *SILV *in a collection of non-pigmented bovine tissues**. A: *SILV *RT-PCR products generated with primers spanning exon 5 to exon 7 (E5_F1 _– E7_R1_) in RNA from non-pigmented tissues; sk: skin, pg: pituitary gland, tg: thyroid gland, ki: kidney, ag: adrenal gland, li: liver, lu: lung, he: heart, br: brain, ru: rumen, if: intestinal fat, pf: perirenal fat, sf: subcutaneous fat, mg: mammary gland, du: duodenum, co: colon, je: jejunum, mu: skeletal muscle. B. RT-PCR products generated with GAPDH primers in different non-pigmented tissues.

RT-PCR revealed a specific expression of the bovine *SILV *gene in all 17 investigated tissues from an adult individual: pituitary gland, thyroid gland, kidney, adrenal gland, liver, lung, heart, brain, rumen, intestinal fat, subcutaneous fat, perirenal fat, mammary gland, duodenum, colon, jejunum, skeletal muscle. All three *SILV *primer combinations tested spanning exon 1 to exon 5 (E1_F3 _– E5_R1_), exon 5 to exon 7 (E5_F1 _– E7_R1_) and exon 9 to exon 11 (E9_F1 _– E11_R2_) confirmed this observation (Table 2). The relative amount as indicated by semi-quantitative RT-PCR differed between tissues: a strong *SILV *expression equivalent to skin was seen in a number of tissues with very divergent functions, e.g. thyroid gland and colon, whereas *SILV *was only weakly expressed in brain, muscle and fat tissues (Figure 5). Analysis with primers spanning exon 5 to exon 7 (E5_F1 _– E7_R1_) revealed at least two PCR fragments for all tissues with a pattern similar to that obtained for differentially pigmented skin tissues.

**Figure 6:**
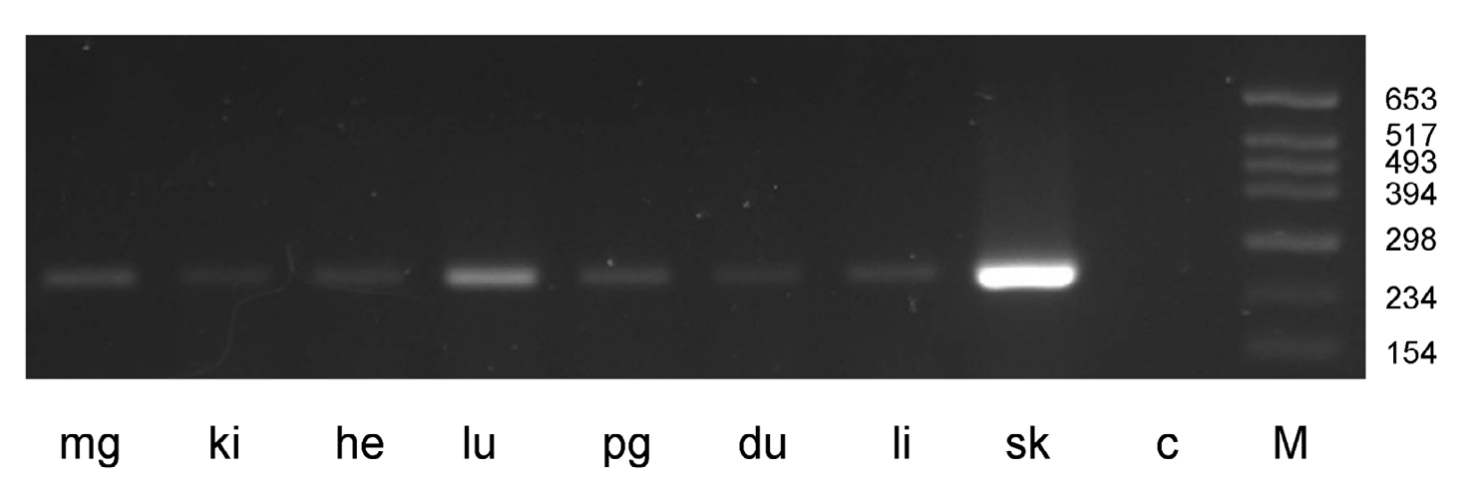
**Splice site specific *SILV *RT-PCR in non-pigmented bovine tissues**. Detection of *SILV *splice variant Δ816–1340 by splice site specific RT-PCR with primers SPex6A_F1 _– E9_R2 _in RNA from non-pigmented tissues. mg: mammary gland, ki: kidney, he: heart, lu: lung, pg: pituitary gland, du: duodenum, li: liver, sk: skin. M: marker; C: negative control

While alternative splice variants were rather uniformly distributed across the differentially pigmented skins, substantial variation was seen for the other tissues (Table 4). Only transcript variant Δ105–362 was tissue-specific for skin. Whereas splice variant Δ816–1340 could be detected in all seven tissues tested (Figure 6), Δ1185–1340 was only found in mammary gland at a low level.

## Discussion

### Structure of the bovine *SILV *gene

The bovine *SILV *gene shows a strong structural homology with the respective gene in human and mouse. The bovine mRNA (GenBank: EF065525) is separated into 11 exons and revealed a homology of 87% to the human (NM_006928.3) and 83% to mouse mRNA (NM_021882.4). At protein level, the similarities were 78 or 73%, respectively. The sharp peak of transcription start sites detected in CAP finding RACE experiments and the putative TATA box of the *SILV *promoter located 30 bp upstream correspond to the classical pattern of TATA box promoter architecture characteristic for genes highly conserved in evolution [24]. Our RACE experiments in the eumelaneic (black) non-dilute (*Dc*^*d*^*/Dc*^*d*^) skin yielded essentially an identical transcription start compared to a *SILV *mRNA obtained by 5'RACE from a homozygous dilute (*Dc*^*D*^*/Dc*^*D*^), crème-white skin [18]. Thus, the use of alternative transcription starts can be formally excluded as the background for differences in coat color between dilute and non-dilute individuals.

### *SILV *transcription in phaeomelaneic and non-pigmented tissues

Taking advantage of our animal model with individuals expressing exclusively eumelanin or phaeomelanin, our RT-PCR results prove that in cattle *SILV *expression in skin is not restricted to areas with eumelanocytes, because we also found *SILV *transcripts in phaeomelaneic and non-melaneic skin. This result is in contrast to previous reports in mice [17] indicating that *SILV *expression could only be detected in cells synthesizing eumelanin. Whereas there are many studies about the melanophore-specific expression of *SILV *in the literature (reviewed by [4]), only one report in humans also describes *SILV *to be ubiquitously expressed [25]. This result is supported by 112 ESTs isolated from non-pigmented tissues in a total of 785 human *SILV *ESTs listed in the NCBI Unigene database [26]). Although no conclusion regarding *SILV *transcription can be drawn from bovine Unigene data base entries, our own experiments clearly reject the hypothesis that *SILV *expression is specific to eumelanocytes in cattle. Expression outside of pigmented tissues indicates that products of the bovine *SILV *gene seem to have a new, up to now unknown function additional to fibril formation in the course of melanosome development. Chakraborty et al. postulate a catalytic role of SILV in melanin synthesis [27]. But as melanin synthesis is restricted to melanophores in skin, uvea and other pigmented tissues [28], involvement of SILV in melanin synthesis would not explain *SILV *expression in tissues without melanophores. Consequently, the additional role of *SILV *expression postulated from our study has to exceed melanophore specific functions. Up to now, it is completely unclear, which role *SILV *transcription may play in e.g. thyroid gland or colon, two tissues with extremely different cell type composition, for which a high *SILV *expression was detected in our experiments. If indeed human *SILV *expression is also not restricted to melanophores as indicated by [25], cattle may serve as an appropriate model to investigate its potential function in non-melanophores.

### Alternative splicing in *SILV *affecting functionally relevant domains

RT-PCR with an overlapping panel of primer pairs indicated nine alterative splice sites in the bovine *SILV *gene resulting in splicing of cryptic introns predominantly affecting exon 6, but also exons 2, 3, 4, 5 and exon 9. Thus, the splicing pattern comprises the use of single or combined cassette exons and alternative 5' splice sites resulting predominantly in deletions of single or multiple exons, which is in line with other comprehensive studies investigating alternative splicing [21]. Sequence alignment of the bovine *SILV *cDNA with the nine cattle ESTs homologous to *SILV*, which are deposited in the Unigene database [26], did not indicate alternative splicing. However, the confirmed alternative *SILV *transcripts in cattle from our study are in line with previous experiments in humans describing two alternative splice sites in exon 6 and exon 9 of the *SILV *gene [19]. The extent of alternative splicing seen in cattle exceeds the number of published alternative transcripts in humans substantially, and we even cannot exclude that further splice variation is present in cattle. The Alternative Splicing Database [29,30] collecting electronic data on constitutive and alternative transcripts lists entries exclusively for human *SILV *and indicates that the number of alternative transcripts in humans is also presumably higher than presently considered. Interestingly, humans and cattle are the only species for which alternative transcripts have been experimentally established [19,29]. No additional transcripts are published e.g. in mice.

Comparative analysis of the bovine SILV protein with the respective counterpart in human according [4] suggested that several functional domains of the SILV protein as defined by [31] should be affected by alternative splicing in cattle. Splice variant Δ105–362 characterized by the complete skipping of exons 2 and 3 results in loss of the proximal part of the N terminal domain (NTD) of the bovine SILV protein. Thus, skipping of exons 2 and 3 would affect the posttranslational modification of the protein substantially, because the NTD carries the majority of SILVs' glycosylation sites as reviewed by [4]. The alternative transcripts Δ363–1340, Δ498–1340 and Δ660–1340 lack 326, 281 or 227 amino acids of the constitutive transcript, respectively, representing the C terminal part of the N terminal domain and the entire Polycystic kidney disease (PKD) and Repeat (RPT) domains. While the PKD has an immunoglobulin folding structure and is thought to mediate protein-protein interactions, the RPT domains seems to be necessary for fibril formation in melanosomes [31]. The entire RPT domain is also missing in the Δ816–1340 transcript in addition to the distal PKD domain. Transcript Δ1185–1340 lacks the distal part of the RPT domain, whereas Δ1332–1340 is devoid of 3 amino acids of the Gap2 domain. Splice variant Δ1655–1748 is the only transcript with a disrupted reading frame and a premature stop codon generating a truncated protein without transmembrane and C terminal domain or possibly inducing a nonsense-mediated mRNA decay [32]. While the Kringle-like domain and the transmembrane domain seem to be rather invariable, the RPT domain is most frequently affected by the different splice variants in our data set. Experiments with human *SILV *deletion constructs showed that a protein lacking the Repeat domain (ΔRPT SILV) showed an appropriate intracellular trafficking [31]. However, HELA cells overexpressing ΔRPT SILV formed only abnormal fibrils, indicating that the RPT domain plays a crucial role in the development of the striated fibrillar structure in melanosomes. Hoashi et al. [31] used HMB45, an antibody frequently employed for melanoma diagnostic as a probe specific for melanosomal fibrils and showed that its epitope is located in the second and third amino acid repeat of the RPT domain. Thus, bovine transcripts lacking the respective part of the RPT domain (like 498–1340, Δ660–1340 and Δ816–1340 in our study) should not be able to form intact fibrils, a process central to appropriate melanosome development as determined for human SILV. Interestingly, the two of the four isoforms of SILV in humans due to alternative splicing also affect the RPT domain albeit not the epitope for HMB45 [31]. The alternative splice variants lacking entire functional domains as confirmed for the bovine *SILV *gene represent ideal candidates for further study into the potential effects of alternative splicing on function [21].

### Variation in alternative splicing of *SILV *with respect to coat color phenotype

In cattle, we did not find any mutation in the coding *SILV *sequence that could convincingly be associated with phaeomelanin coat color dilution in a F_2 _resource population based on the Charolais and the Holstein breed [14]. In dogs, Clark et al., [12] described a mutation in intron 10 of the canine *SILV *gene that was associated with coat color dilution and concluded that the mutation might impair correct splicing of the canine *SILV *transcript. Thus, another as yet undetected mutation in the genomic sequence of the bovine *SILV *gene could possibly affect the structure of the *SILV *mRNA, representing the genetic background for phaeomelanin dilution in cattle. Because we could confirm *SILV *expression in eumelaneic and also phaeomelaneic skin, any genetic variant affecting regulation or coding structure of the gene might theoretically also affect phaeomelanin dilution in cattle, although presently there is no indication, which function SILV may exert on maturation of phaeomelanosomes. Thus, given our expression data the *SILV *gene could not formally be rejected as the background for phaeomelanin dilution in cattle. However, neither the transcription start, which was conserved between homozygous dilute (*Dc*^*D*^*/Dc*^*D*^) and non-dilute (*Dc*^*d*^*/Dc*^*d*^) individuals, nor the distribution of splice variants in the differentially pigmented skins, convincingly explained the differences in dilution phenotype. Hence, there is no indication that variation in the primary sequence of the SILV protein either due to variation in the coding sequence or due to alternative splicing is responsible for dilution of phaeomelanin in cattle.

Whereas the pattern of transcript variants is rather similar across the panel of differentially pigmented skin, the variability of splice variants detected in other tissues points towards a tissue specific splicing mechanism. This may represent a tool for adapting *SILV *expression to the requirements of the respective cells [20].

## Conclusion

Although the structure of the *SILV *gene is conserved across a variety of species including cattle, its pattern of transcription shows substantial differences regarding alternative splicing between cattle and human on the one side and mice on the other. Our experiments provide evidence for a ubiquitous transcription of the bovine *SILV *gene not restricted to pigmented cells and show a striking variety of alternative splice sites. These results indicate that potentially *SILV *may have functions exceeding melanosome development. This would have to be considered in future investigations concerning melanoma diagnostic and therapy and also in studies taking SILV as a model for amyloid formation [33] or for intracellular transport mechanisms. The similarity in expression pattern compared to human and the variety of alternative transcripts predestine bovine *SILV *as an adequate animal model.

Whereas alternative splicing is a well established regulatory mechanism for gene expression [20], the confirmed alternative *SILV *transcripts are in line with the hypothesis of the new postulated additional *SILV *functions exceeding synthesis and deposition of melanin. This hypothesis is supported by the detection of *SILV *transcripts in non-melanophores in our study. However, it has to be considered that our analyses are restricted to the transcription level. Thus, subsequent steps modulating *SILV *expression due to e.g. nonsense mediated decay [32] and translational and posttranslational modification require further investigation to confirm the postulated additional functions of the *SILV *gene. Due to its extensive, up to now unique pattern of alternative splicing, bovine splice variants represent a naturally occurring model for the function of the domains located in the spliced regions of the *SILV *and for the alternative functions postulated for *SILV*. Furthermore, factors regulating the processes of alternative splicing in the different tissues can be investigated exemplarily.

## Methods

### Tissue samples

For our study, we included individuals from a Charolais × German Holstein F_2 _cattle resource population [34]. The resource population segregates for the coat color trait loci (i) *Dilution *(*Dc*) responsible for coat color dilution, (ii) *Extension *(*E*) responsible for an eumelaneic (black) or a phaeomelaneic (red) phenotype, and (iii) *Spotted *(*S*) resulting in a pigmented-white spotting pattern similar to piebald in mice. We collected differentially pigmented neck skin after slaughter: (1) black and white sections of a homozygous non-dilute (*Dc*^*d*^*/Dc*^*d*^), spotted (*s/s*) eumelaneic (*E*^*D*^*/E*^*e*^) animal, (2) diluted colored and white sections of an eumelaneic (*E*^*D*^*/E*^*D*^), heterozygous *Dc*^*D*^*/Dc*^*d*^, spotted (*s/s*) individual, (3) diluted colored and white sections of a phaeomelaneic (*E*^*e*^*/E*^*e*^), heterozygous *Dc*^*D*^*/Dc*^*d*^, spotted (*s/s*) individual and (4) the crème white skin of a homozygous non-dilute (*Dc*^*D*^*/Dc*^*D*^), non-spotted, eumelaneic (*E*^*D*^*/E*^*D*^) individual. Hair and subcutaneous tissue was removed and the remaining cutis was snap frozen. Genotypes of the individuals for the *SILV *c64G>A mutation, presumably responsible for dilution of eumelanin, were determined as described by [14]. Genotypes at the dominant-recessive *Extension *locus were determined by sequencing of the MC1R gene. Primers MC1R_F1 _(5'-TACTACTTTATCTGCTGCCTG-3') and MC1R_R1 _(5'-GCGTAGAAGATGGAGATGTAG-3') flanking the causal mutations for the alleles *E*^*D *^(dominant black, eumelanin) and *E*^*e *^(recessive red, phaeomelanin) were used for amplification in genomic DNA of individuals tested and consecutive sequencing of the resulting PCR products. In addition to the differentially pigmented skin samples, 17 tissues (pituitary gland, thyroid gland, kidney, adrenal gland, liver, lung, heart, brain, rumen, intestinal fat, subcutaneous fat, perirenal fat, mammary gland, duodenum, colon, jejunum, skeletal muscle) from an adult female Charolais × German Holstein F_2 _individual were collected at slaughter and immediately snap frozen. Total RNA was isolated with the NucleoSpin^® ^RNAII kit (Macherey and Nagel) except all fat tissues, which were isolated with the RNeasy Lipid tissues kit (Qiagen), essentially as described by the manufacturers.

### Analysis of skin *SILV *gene expression

The isolated RNA was reverse transcribed into cDNA according to [35] with a primer mix containing Oligo dT_(12–18) _and a gene specific primer from the 3'UTR of the SILV mRNA (E11_R2_, Table 1). The resulting cDNAs were amplified with GO-Taq polymerase (Promega) under standard conditions and primers as indicated in Table 1 and Table 2. Due to confirmed splice variants in the RPT and in the GAP3 domain of the human *SILV *gene and because we obtained suggestive additional bovine *SILV *transcripts in initial investigations, the entire bovine *SILV *cDNA was examined for additional splice sites using overlapping PCR primer combinations (Table 2). These investigations were carried out in skin of different coat color phenotypes. The generated PCR fragments were separated on agarose gels. As a control, expression of glyceraldehyde-3-phosphate dehydrogenase (*GAPDH*) was determined by RT-PCR as described [35].

### Sequencing of *SILV *transcripts

The analysis of *SILV *transcripts in differently pigmented skin and non-pigmented body tissues detected more than one RT-PCR fragment for several primer combinations. The respective fragments were excised from the agarose gel, purified using the Nucleospin Extract II kit (Macherey and Nagel) and sequenced on a capillary sequencer (ABI 310, Applied Biosystems; MEGABACE, GE Healthcare) using BigDye^® ^(Applied Biosystems) chemistry. Two prominent RT-PCR fragments > 1000 bp generated with primers spanning exon 1 to exon 11 (5UTR_F1 _– E11_R2_, Table 1) were excised from the gel, purified, and cloned into the pCR4 Blunt TOPO vector (Invitrogen) according the manufacturers instructions. Seven size fractionated subsets of RT-PCR fragments generated by primers enclosing exon 1 to exon 7 (E1_F3 _– E7_R1_, Table 1) were excised from the agarose gel, purified and cloned into pDrive vector (Qiagen) according to the manufacturers instructions. Clones with different insert sizes according to colony PCR were sequenced on a capillary sequencer (ABI 310, Applied Biosystems; MEGABACE, GE Healthcare) using BigDye^® ^(Applied Biosystems) chemistry.

### *SILV *RNA 5' RACE experiments

To determine the transcription start site for the bovine *SILV *gene and to test for alternative promoters, 5' RACE experiments with the GeneRacer Kit (Invitrogen) based on RNA ligase mediated and oligo-capping methods were performed. After CAP selection in a preparation of total RNA from black skin of a eumelaneic (*E*^*D*^*/E*^*D*^), non-dilute (*Dc*^*d*^*/Dc*^*d*^) individual, reverse transcription with oligo dT and re-amplification with the RACE 5' oligo and a *SILV *specific primer from exon 7 (RACE_E7N, Table 1) was carried out according to the manufacturers instructions. The generated fragments were cloned into pDrive vector using the Qiagen PCR cloning kit (Qiagen). Clones with different insert sizes according to colony PCR were sequenced. Sequences obtained were aligned to the deposited *SILV *mRNA (GenBank: EF065525).

### Confirmation of *SILV *splice variants

To confirm the detected *SILV *splice variants, *SILV *cDNA samples were investigated by splice site specific RT-PCR. For this purpose, *SILV *cDNAs were generated by reverse transcription with the E11_R2 _primer from total RNA of skins differing in coat color phenotype. Afterwards, the cDNAs were subjected to PCR amplification with one *SILV *primer corresponding to the sequence of the constitutive *SILV *mRNA and another primer specific for each splice variant, respectively (Table 1, Table 3). Splice variant specific primers were designed to bridge the splice ends of the addressed alternative splice site. For negative control, DNA of a plasmid with a constitutive *SILV *cDNA insert was included.

### *SILV *expression in non-pigmented tissues

Finally, *SILV *expression was screened in a collection of 17 total RNAs from non-pigmented tissues (pituitary gland, thyroid gland, kidney, adrenal gland, liver, lung, heart, brain, rumen, intestinal fat, subcutaneous fat, perirenal fat, mammary gland, duodenum, colon, jejunum, skeletal muscle). Analogous to the RT-PCR in skin tissue, the different RNAs were reversely transcribed and amplified by using primers corresponding to the constitutive *SILV *cDNA sequence in combinations covering exon 1 to exon 5 (E1_F3_/E5_R1_), exon 5 to exon 7 (E5_F1_/E7_R1_) and exon 9 to exon 11 (E9_F1_/E11_R2_, Table 2).

Because there was evidence on alternative splicing in non-pigmented skin, a collection of eight non-pigmented other tissues (liver, duodenum, pituitary gland, lung, heart, kidney, mammary gland) was further investigated for alterative splicing by splice variant specific RT-PCR analogous to the procedure described for skin. The respective primer combinations are listed in Table 3.

## Competing interests

The author(s) declares that there are no competing interests.

## Authors' contributions

CK conceived, designed and coordinated the study, carried out PCR analysis of RNAs, participated in sequence alignments and drafted the manuscript. RW participated in coordination of the study, participated in sequence alignments, carried out the RACE and splice variant specific experiments, and helped drafting the manuscript. All authors read and approved the final manuscript.
